# Influence maximization under the linear threshold model on a CMOS Ising solver

**DOI:** 10.1038/s41598-025-27169-5

**Published:** 2025-12-07

**Authors:** Ziqing Zeng, S. Ramprasath, Hüsrev Cılasun, Abhimanyu Kumar, Chris H. Kim, Ulya R. Karpuzcu, Sachin S. Sapatnekar

**Affiliations:** 1 University of Minnesota, Minneapolis, USA; 2 Indian Institute of Technology Madras, Chennai, India

**Keywords:** Electrical and electronic engineering, Computer science

## Abstract

Influence Maximization (IM) is a fundamental problem in network science with applications in viral marketing, information dissemination, cybersecurity, and epidemiology. Classical IM solvers often trade off solution quality for runtime efficiency due to the NP-hardness of typical models such as Linear Threshold, Independent Cascade, and Triggering. Among these, the Linear Threshold model stands out by avoiding costly Monte Carlo simulations, enabling a more tractable Ising formulation. In this study, we propose a novel workflow for solving the Linear Threshold IM problem on directed acyclic graphs using a CMOS Ising solver through an Ising formulation. Our approach combines an integer linear programming-based Ising formulation with hardware-aware decomposition and preprocessing steps that adapt the model to the constraints of the hardware solver. We evaluate the efficiency of our approach under various coefficients and identify the optimized configuration. Experimental results show that our approach achieves superior solvability over some state-of-the-art IM solvers while maintaining competitive runtime and orders-of-magnitude lower energy consumption in both randomly generated and real-world benchmarks. These results demonstrate the potential of Ising solvers for energy-efficient IM applications.

## Introduction

Influence maximization (IM) identifies a small set of individuals or nodes, called the *seed set*, to maximize the spread of influence across a social network. Due to its commercial value, it has been widely studied, especially in the context of information diffusion: for example, in viral marketing, companies seek to find a seed set of influencers whose social connections can promote a product^1–3^. Similarly, IM is also applied in network monitoring, rumor control, and recommendation systems^4^.

Diffusion models have been used to describe the spread of influence in IM. These models simulate the activation of other individuals from the seed set, based on rules of behavior and interaction^2,4,5^. Widely-used IM models include the linear threshold (LT), independent cascade (IC), triggering (TR), and continuous-time (CT). The LT model^6,7^ activates a node when the aggregate influence from its neighbors surpasses a threshold, while the IC model^2,8^ allows each node to activate its neighbors with a certain probability independently. The TR model^2^, which generalizes IC and LT, chooses a random triggering set for a node from its neighbors according to a predefined distribution, such that the node becomes active if any node in its triggering set is activated. The CT model^5^ captures the time dynamics of influence propagation, with information propagating between nodes based on a continuous distribution over time, and with each edge having an associated propagation rate or probability.

Each IM model is tailored to capture different types of interactions and diffusion processes. Under all of the models mentioned above—IC, LT, TR, and CT—the IM problem is NP-hard^2,4^. Classical heuristics for solving these formulations can be classified into three categories^4^: (i) simulation-based algorithms rely on Monte Carlo (MC) simulations to estimate the influence spread of seed sets; (ii) proxy-based algorithms use approximate methods or proxies to estimate influence spreads, bypassing the complexity of direct computations; and (iii) sketch-based algorithms use compact data representations or “sketches” to estimate influence spreads more efficiently while maintaining approximation guarantees.

The quantum-inspired Ising formulation offers a promising alternative for solving the IM problem. The Ising model is derived from the mathematical representation of magnetic interactions, where the system seeks to reach a minimum-energy configuration. This approach has been used to address many combinatorial optimization problems (COPs), such as min-cut, max-cover, and 3-SAT^9–11^. These COPs can be written in Ising form via quadratic unconstrained binary optimization (QUBO) formulations and then mapped to a hardware Ising solver^11^. Under the IC model, the IM problem can be mapped to a maximum coverage problem via reverse influence sketch methods, where the randomly generated reverse reachable sets serve as a Monte Carlo simulation to model the stochastic diffusion process. This maximum coverage problem is then solved effectively with Ising solvers^12^. The IC-model-based Ising formulation^12^ requires expensive Monte Carlo simulations to determine influence, but the LT model, which does not require Monte Carlo simulations in the optimization loop, can provide a better Ising model where the Ising solution can be directly interpreted into the IM solution.

In particular, since numerous networks of interest can be represented as directed acyclic graphs (DAGs), the problem of IM on DAG structures is of great interest. Prior work has applied the LT model of IM to DAGs, referred to as the linear threshold directed acyclic graph (LDAG) influence model, has shown this to be NP-hard^7^. In this paper, we propose a solution for the LDAG model, going from an Ising formulation of the problem to its solution on COBI^13^, a hardware Ising solver with all-to-all coupling, implemented in a low-power CMOS chip. Our approach first represents the problem using a constrained ILP formulation. Our ILP draws ideas from two prior solutions: Ackerman et al.^14^ solve a simplified 0-1 ILP formulation without edge weights that is shown in later work^15^ to be applicable only to unweighted DAGs, and Baghbani et al., which creates a general non-0-1 ILP for a directed graph with cycles, but requires multiple variables per vertex, including the activation episode to represent the timestep at which the node is activated by its neighbors. In contrast, our approach handles weighted DAGs and requires only one activation variable per vertex.

This ILP is then translated into an unconstrained QUBO formulation, which is used to derive the Ising formulation via a variable transformation. Next, this formulation must be mapped to the Ising hardware chip, which imposes a set of restrictions: specifically, a limited number of spins and limited coupling strength precision. We use problem decomposition methods to overcome the former issue, and use scaling and clamping methods for the latter problem, and show how the LDAG influence model can be effectively solved on Ising hardware.

The contributions of this paper include: (1) an ILP formulation for the LT model that is compatible with Ising solvers; (2) a novel hardware-aware hybrid workflow that integrates graph decomposition and preprocessing to enable efficient IM solution on the COBI chip; (3) a detailed evaluation of the performance of a COBI-based workflow on IM benchmarks, demonstrating improvements in energy efficiency and solution quality compared to classical IM solvers and a software-based Ising solver.

## Linear threshold IM formulations

### The linear threshold influence model for IM

IM was initially formulated as an algorithmic problem^1,2^, focused on analyzing a social network graph $G = (V, E)$, where *V* is the set of vertices (or nodes), representing individuals, and *E* is the set of directed or undirected edges, representing the social links between individuals. In a directed graph, an edge direction indicates the direction of influence. Each node in the graph is assigned an active or inactive binary status. Initially, all nodes are inactive. To determine the influence of a seed set, $S \subseteq V$, the nodes in this set are first activated. Next, through the diffusion process *M*, the seed nodes in *S* may activate their neighbors; the newly activated nodes can further activate their neighbors; and so on. When the diffusion process terminates, the number of active nodes corresponds to the influence of the seed set. Under the progressive model, activated nodes cannot be deactivated.

IM identifies the set of the most influential nodes as the seed set, to maximize the influence of the diffusion process across the graph. Denoting the influence spread function, which quantifies the total number of active nodes in the diffusion from seed set *S* as $\sigma _{G,M}(S)$, the goal of IM is to maximize the influence to find the influence set $S^*$ of the largest cardinality, i.e.,

1 $$\begin{aligned} \textstyle S^*={{\,\textrm{argmax}\,}}_{S\subseteq V \wedge |S|= k}\sigma _{G,M}(S) \end{aligned}$$

Under the LDAG model, the model *M* corresponds to LT, and the graph *G*(*V*, *E*, *W*) is a DAG, where each edge $e=(u,v) \in E$ is associated with a nonzero weight, $w(u,v) \in W$. We assume that all the edge weights and thresholds are normalized, i.e., the sum of all incoming edge weights into *v* satisfies:

2 $$\begin{aligned} \textstyle \sum _{u\in N_{in}(v)}w(u,v) \le 1, \end{aligned}$$

where $N_{in}(v)$ is the set of incoming edges of node *v*. The LT model defines the diffusion mechanism that causes a node to switch its status from inactive to active. We define an edge weight as active if its originating node has been activated.

During diffusion, initially, only nodes in the seed set are activated. Denoting the set of activated nodes in step *i* as $A_i$, implies that $A_0 = S$. At each subsequent step, an inactive node *v* is activated if and only if the sum of edge weights of all active incoming edges to node *v* is no less than a node-specific threshold, $\tau _v$. Thus, LT diffusion is summarized as:

3 $$\begin{aligned} \textstyle A_0 = S; \quad A_i = A_{i-1} \cup v, \; v\in V\backslash A_{i-1} \; \text {if and only if} \sum _{u\in \cup _{0\le j\le i-1}A_j }w(u,v)\ge \tau _{v} \end{aligned}$$

**Fig. 1:**
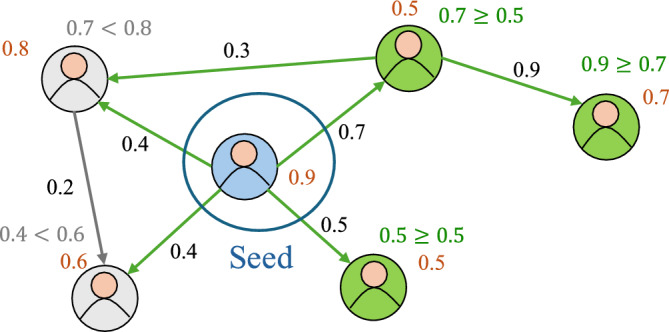
A diffusion graph example under the LT model.

Fig. 1 illustrates an example of diffusion under the Linear Threshold (LT) model. Each node has a threshold shown in red, and the directed edge weights are labeled in black. If the blue node is chosen as the seed, it can activate neighboring nodes (shown in green) over multiple steps, provided the sum of incoming weights from already activated nodes meets or exceeds each node’s threshold. In contrast, gray nodes remain inactive because their incoming weights fall short of the threshold. Identifying the blue node that activates the most green nodes corresponds to solving the IM problem under the LT model with a seed set size of one.

The maximum active set problem is a simplified version of the LT model of IM, where all edges are assumed to be identical and unweighted, and the activation threshold for each node is defined based on the number of incoming active edges rather than their weighted summation. Based on the ILP for the maximum active set problem^14^ for an unweighted directed graph, an ILP formulation for the LT model for IM has been presented by extending the diffusion constraints with weights^15^.

The formulation includes a set of variables to track the activation status of nodes throughout the diffusion process. For cyclic graphs, each node requires multiple variables for its activation episode, to represent the time step at which the node is activated by its neighbors^15^. In contrast, one variable per node is sufficient for DAGs.

To indicate the final activation status of a node *v*, a Boolean variable $y_v$ can be employed, where $y_v = 1$ denotes that the node is active; otherwise, it is inactive. The activation condition (3) can be expressed as a simplified conditional:

4 $$\begin{aligned} y_v = {\left\{ \begin{array}{ll} 1,\quad \text {if}\quad v\in S \\ 1,\quad \text {if}\quad v\in V\backslash S \quad \text {and} \quad \sum _{u\in N_{in}(v)}w(u,v)\ge \tau _{v}\\ 0,\quad \text {otherwise} \end{array}\right. } \end{aligned}$$

The conditions in (4) indicate that all the nodes in the seed set are active; those outside the seed set can be active if they satisfy the activation condition; other nodes are inactive. We will later use relations in the form of  (4) as optimization constraints.

### ILP and Ising formulation of the LDAG influence model

Our ILP formulation for the LDAG problem with edge weights can be stated as follows:

5 $$\begin{aligned} \left\{ {\text {Minimize} \quad -\sum _{v\in V}y_{v}\quad \text {subject to:}} {\left\{ \begin{array}{ll} y_{v}\in \{0,1\} \; \forall v\in V, s_{v}\in \{0,1\} \; \forall v\in V \qquad \qquad \qquad (5a) \\ \textstyle \sum _{v\in V}s_v=k \qquad \qquad \qquad \qquad \qquad \,\qquad \qquad \qquad (5b)\\ s_v\le y_{v} \quad \forall v\in V\qquad \qquad \qquad \qquad \quad \qquad \quad \qquad \quad (5c)\\ y_v-s_v\le \left( \textstyle \sum _{u\in N_{in}(v)}y_{u}w(u,v) \right) / \tau _{v} \quad \forall v\in V\quad \quad (5d)\\ \end{array}\right. } \right. \end{aligned}$$

Here, $y_v$ and $s_v$ are Boolean variables (5a), where $y_v$ is defined in (4) and $s_v$ is set to 1 if node *v* is part of the seed set, and is 0 otherwise. The constraint (5b) ensures that the number of seed nodes (for which $s_v = 1$) must equal the seed set limit. Inequality (5c) ensures that all nodes in the seed set are active, by forcing $y_v = 1$, while (5d) is the condition that determines whether a node is activated during diffusion. Given (5c), $y_v-s_v$ can be 0 or 1. If $y_v-s_v=1$, then $y_v=1$ and $s_v=0$, implying that node *v* is not in the seed set but becomes active, satisfying the activation condition of node *v*. The influence maximization is converted to a minimization problem by reversing the sign of the objective function.

The objective function and constraints (5a)–(5d) represent a constrained optimization problem with binary variables, and we translate it to QUBO form. The binary constraints (5a) are fundamental requirements for QUBO, and the constraints (5b)–(5d) are brought into the QUBO objective through the introduction of a penalty term, weighted by a penalty coefficient^16^.

To formulate the complete QUBO objective function, penalty coefficients $\lambda _1$, $\lambda _2$, and $\lambda _3$, are applied to all constraint-based terms, which are then added to the original objective function. The full QUBO objective function is expressed as:

6 $$\begin{aligned} \min f(s_v,y_v,b_v,f_{v,i})&= -\sum _{v\in V}y_v + \lambda _1\sum _{v\in V} (s_v-k)^2 + \lambda _2\sum _{v\in V}(y_v-s_v-b_v)^2 \nonumber \\ &\quad + \lambda _3 \sum _{v\in V} \left( \sum _{(u,v)\in E}y_u\cdot w(u,v)-\tau _v\cdot (y_v-s_v)-\sum _{i} f_{v,i} c_i \right) ^2 \end{aligned}$$

**Table 1:** IM constraints and their corresponding terms in the objective function.

Constraints	Term in objective function
$\sum _{v\in V}s_v=k$ (5b)	$(\sum _{v\in V}s_v-k)^2$
$s_v\le y_{v} \quad \forall v\in V$ (5c)	$\sum _{v\in V}(y_v-s_v-b_v)^2$
$y_v-s_v\le \frac{\sum _{u\in N_{in}(v)}y_{u}w(u,v)}{\tau _{v}} \quad \forall v\in V$ (5d)	$\sum _{v\in V}(\sum _{(u,v)\in E}y_u\cdot w(u,v)-\tau _v\cdot (y_v-s_v)-\sum _{i} f_{v,i} c_i )^2$

Table 1 illustrates the process of translating these constraints into terms within the QUBO objective function. As an equality constraint, (5b) can be directly incorporated as a squared term in the QUBO objective function. Constraints (5c) and (5d) are inequalities, and slack variables ($b_v$ and $f_{v,i}$, respectively) are required to convert them to equalities to facilitate their transfer into the objective function. Since the threshold $\tau _v \le 1$ is a real number, the slack in (5d) is not binary; we represent it using multiple bits, $f_{v,i}$, weighting each bit with a positional constant $c_i = 2^{-i}, i \in [1,N_w]$, where $N_w$ is the number of binary bits of precision. Thus, $\sum _{i} f_{v,i} c_i$ covers fractional values between 0 and 1. Thus, $(P(v)-\sum _{i} c_if_{v,i})^2$ in the objective function is equivalent to $P(v) \ge 0$ for any $P(v)<1$, where *P*(*v*) can be replaced with appropriate terms from (5d). The number of binary spin variables, $f_{v,i}$, for each node *v* is determined by the precision, i.e., the least significant fraction (LSF) in the weights.

The purpose of introducing the $\lambda _i$ values is to ensure that there is a sufficient penalty, such that the optimizer does not benefit from violating the constraints defined in Eqs. (5a)–(5d) during the minimization of $-\sum _{v\in V}y_v$. Once a constraint is violated, the penalty should always exceed any possible improvement gained in the objective function. In a graph with *N* nodes and $N_w$ bits in weight precision, in (6), the minimal penalty for the quadratic terms associated with $\lambda _i$ are 1, 1, and $4^{N_w}$. Based on the worst-case assumption, the minimal theoretical $\lambda _i$ values are:

7 $$\begin{aligned} \lambda _1 = \lambda _2 = N, \quad \lambda _3 = 4^{N_w}\cdot N \end{aligned}$$

Due to the limitations of the Ising hardware, there are practical trade-offs in the choice of these values, as discussed in “Formulation exploration”.

In addition, in the process of minimizing the objective function in (6), we can eliminate the slack variables $b_v$ by simplifying the penalty term involving $\lambda _2$. As illustrated in Table 2, the expression $\min _{b_v}{(y_v - s_v - b_v)^2}$ has the same truth table as $s_v - y_v s_v$ for all combinations of $s_v$ and $y_v$. Thus, one can be substituted for the other.

**Table 2:**
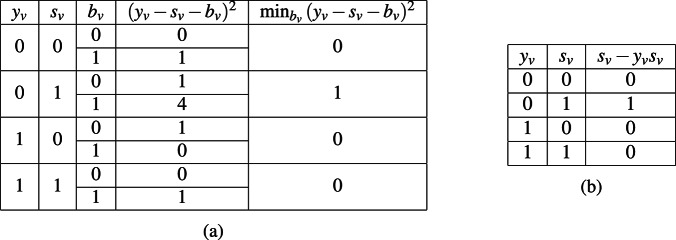
Truth table for constraints terms (a) $\min _{b_v}{(y_v-s_v-b_v)^2}$, (b) $s_v-y_v s_v$.

We introduce the function $g(s_v,y_v,f_{v,i})$ to represent the terms that do not involve $\lambda _2$ and $b_v$ in (6). Therefore,

8 $$\begin{aligned} \min f(s_v, y_v, b_v, f_{v,i})&= \min _{s_v, y_v, b_v, f_{v,i}} \{ g(s_v, y_v, f_{v,i}) + \lambda _2 \sum _{v \in V} (y_v - s_v - b_v)^2 \}\nonumber \\&= \min _{s_v, y_v, f_{v,i}} \{ g(s_v, y_v, f_{v_i}) + \lambda _2 \sum _{v \in V}(s_v-y_vs_v) \} \nonumber \\&= \min _{s_v, y_v, f_{v,i}} \{ -\sum _{v\in V}y_v +\lambda _1(\sum _{v\in V}s_v-k)^2 + \lambda _2\sum _{v\in V}(\varvec{s_v-y_vs_v}) \nonumber \\ &\quad + \lambda _3 \sum _{v\in V}(\sum _{(u,v)\in E}y_u\cdot w(u,v)-\tau _v\cdot (y_v-s_v)-\sum _{i} c_if_{v,i})^2 \} \end{aligned}$$

The second equality, which is free of $b_v$, arises from the observation above based on Table 2. In subsequent sections of this paper, we will refer to the QUBO objective function (8) for the LDAG IM model as the standard QUBO formulation.

In summary, the spins involved in the QUBO formulation of the LDAG influence model consist of $s_v$, $y_v$, and $f_{v,i}$. The cardinalities of $s_v$ and $y_v$ equal the number of nodes in the graph. To account for fractional values between zero and one, it is necessary to include $\lceil -\log _2\text {LSF} \rceil$ binary variables for $f_{v,i}$. Consequently, the total number of required QUBO variables in the standard QUBO formulation is expressed as $(2+\lceil-\log_2\text {(LSF)}\rceil)N_V$ in total, where $N_V$ denotes the number of nodes.

## A CMOS hardware Ising solver

### QUBO/Ising formulation

A QUBO problem with n variables is formulated as minimizing a Hamiltonian objective function as:

9 $$\begin{aligned} \textstyle \min _{{\textbf{x}}} F({\textbf{x}}) = {\textbf{x}}^T Q {\textbf{x}} = \sum _{i=1}^n Q_{ii} x_i + \sum _{i=1}^n \sum _{j=1, j \not = i}^n Q_{ij} x_i x_j, \end{aligned}$$

where ${\textbf{x}}$ is a $1\times n$ boolean vector as variable and *Q* is an $n\times n$ matrix containing the coefficients of the QUBOs problem. The diagonal elements $Q_{ii}$ multiply $x^2 =x_i$ since $x_i \in \{0,1\} \forall i$. Setting $x_i=(s_i+1)/2$ in Eq. (9) we obtain the Ising formulation:

10 $$\begin{aligned} \textstyle \min _{{\textbf{s}}} F({\textbf{s}}) = \sum _{i=1}^{n} h_i s_i + \sum _{i=1}^n \sum _{j=1, j \ne i}^n J_{ij}s_i s_j, \end{aligned}$$

where each Boolean variable $x_i$ is converted into a spin variable $s_i \in \{-1,+1\}$. This transformation allows any QUBO problem to be expressed in Ising form, compatible with quantum and classical annealing solvers, where $J_{ij}$ and $h_i$ are coupling weights.

### Hardware Ising solver

**Fig. 2:**
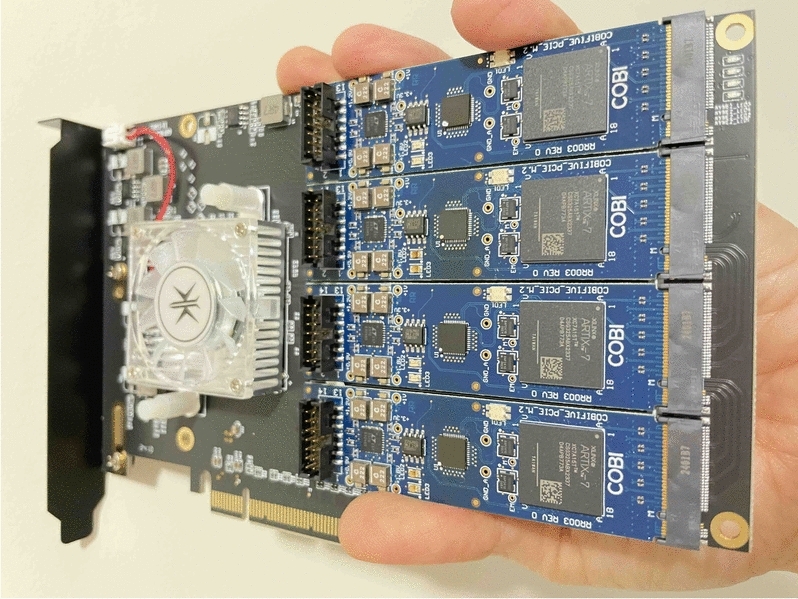
A PCIe card with the CMOS-based COBI hardware Ising solver.

**Table 3:** Hardware specifications of the COBI solver chip.

Name	COBI
Technology	28nm
Chip size	4mm $\times$ 4mm
# of RO spins	46
Connectivity	All-to-all
Coupling weights	$h_i,J_{I,j} \in {\mathbb {Z}}; \; -14 \le h_i, J_{I,j} \le 14$
AXI clock	50MHz
Ising core time	164$\mu$s
Overall runtime (Python package runtime)	$\sim$400$\mu$s
Power	24mW

We overview the coupled oscillator-based Ising solver (COBI) hardware solver^13^ that is employed to solve the IM problem. Unlike prior approaches based on technologies that draw large amounts of power and must be supercooled to milliKelvin levels, COBI uses mainstream CMOS technologies that are easily manufacturable, operate at room temperature, and consume low power^17^.

COBI utilizes the dynamics of coupled ring oscillators to obtain solutions to the Ising model. Each oscillator phase, ranging $[0, 2\pi )$, represents a spin, while interactions between oscillators emulate the energy landscape of the problem.The solution process consists of programming the coupling weights into the ring oscillator array and sampling the phase of each oscillator after synchronization. Due to the physical properties of the oscillators, the system naturally evolves and converges to a low-energy state, with the final phases representing the resulting spin configuration. This hardware-based process is significantly faster than solving Ising formulations using mathematical optimization heuristics on a CPU.

Unlike other approaches that build Ising machines in hardware using nearest-neighbor connections^11,18,19^, COBI employs CMOS ring oscillators to represent spins and has several notable features. First, it offers all-to-all (A2A) connectivity between spins that enables flexible problem formulations: to replicate the capabilities of an A2A solver with *k* spins, nearest-neighbor solvers would require $k^2$ spins. Thus, it is equivalent to a locally connected architecture^19–21^ with thousands of spins^22^. Second, compared to quantum Ising solvers that have stringent refrigeration needs^23^, COBI operates at room temperature. Third, unlike the multi-kW power needs of such Ising solvers, COBI requires 24mW of power, thus providing orders-of-magnitude reduction in power consumption. COBI has previously been demonstrated successfully on the 3SAT problem^11^, and in this paper, we apply it to solve the LDAG IM problem. The COBI PCIe card used in our experiment is shown in Fig. 2. The hardware specifications of the COBI solver chip are listed in Table 3.

Despite its advantages, COBI has several limitations common to other hardware Ising solvers: (1) each COBI chip has a limited number of spins, and (2) while a software solver can use floating-point precision for coupling weights, COBI offers about 5 bits of precision, from $-14, \cdots , +14$. We will show how we overcome these limitations as we address the IM problem.

## Mapping the LDAG influence model to a hardware Ising solver

We now address the practical issues associated with implementing the formulation (8) on COBI. We first overview the workflow of our LDAG IM solver and then describe critical steps in the workflow.

**Fig. 3:**
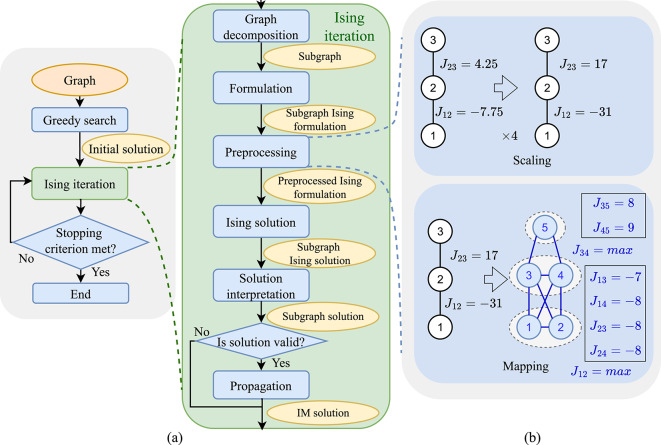
(**a**) Workflow with Ising decomposition and graph decomposition, (**b**) preprocessing techniques.

In the IM solution, either the set of vertices influenced by the seed nodes may be completely disjoint, or they may have a nonempty intersection. For the former case, a greedy search can find the solution. As illustrated in Fig. 3a, the workflow of our IM solver begins with this *greedy search* to account for cases where disjoint clusters yield the optimal influence. In this search, we run a step known as *propagation*, individually from each node. During propagation, we activate the seed node and its influenced nodes, and iteratively compute the overall spread of influence until no further nodes can be influenced. Based on the results of propagation, we greedily select the seeds with the highest influence to form an initial seed set. This serves as the initial IM solution. Next, we perform a series of *Ising iterations*, each with the potential of improving upon the IM solution. The iterations stop once no improvement is observed over a predefined number of consecutive iterations. At that point, the IM solution is considered the final output. An *Ising iteration* in the workflow, described in an inset in the figure, begins with a decomposition of the global graph to extract a subgraph and consists of the following steps:

$\underline{\textit{Graph decomposition}}$ The graph decomposition step aims to overcome issues associated with the limited number of spins on COBI hardware and generates a subgraph whose Ising model can fit on the hardware. In each Ising iteration, the global graph is decomposed into a subgraph using a graph decomposer. One such graph decomposer is the breadth-first search (BFS) decomposer constructed from a BFS traversal^11^. A BFS traversal starts from a source node and visits all its immediate neighbors before moving on to the neighbors of those neighbors, maintaining a queue to track the nodes to be visited. To generate a subgraph with $N_{sub}$ nodes, the BFS traversal is initialized from a random source node and terminates once $N_{\text {sub}}$ nodes have been visited. The subgraph is then defined by these visited nodes, as the output of the BFS decomposer. We propose another graph decomposer, the improved BFS decomposer, which modifies the neighbor selection process during BFS traversal. When visiting one node, instead of adding all its unvisited neighbors into the queue as in the BFS decomposer, the improved BFS decomposer randomly selects at most $\gamma N_{sub}$ neighbors, where $0 < \gamma \le 1$ is a tunable parameter. This selective visitation prevents the generation of subgraphs with shallow depth. All other procedures remain identical to the standard BFS decomposer. A comparison between the BFS and improved BFS decomposers is provided in Figs.S1 and S2 in the Supplementary Materials, including an analysis of the influence of $\gamma$ on performance. Based on these results, we adopt an improved BFS decomposer with $\gamma =0.3$.

$\underline{\textit{Formulation}}$ In this phase, the IM computation on the subgraph is translated into the Ising formulation (8). The choice of the penalty coefficients, $\lambda _i$ is crucial in determining the mapping to hardware. A theoretically sound criterion for choosing their values in (7) assumes that the ideal Ising solver has perfect accuracy and unlimited precision, neither of which is true of real hardware platforms. This results in large penalty coefficients, which may be difficult to implement as they go beyond the maximum precision afforded by the hardware. Moreover, large values may overwhelm the objective function: the hardware Ising solver, which solves the unconstrained optimization problem with the penalty function, may effectively ignore the objective and focus only on satisfying the constraints. However, if the penalty coefficients are smaller, the IM solution provided by the hardware may violate some of the constraints in Table 1. Thus, a practical value of $\lambda _i$ should be neither too large nor too small. We will make suitable choices for the formulation phase in “Formulation exploration”, based on an experimental exploration of the influence of $\lambda _i$.

$\underline{\textit{Preprocessing}}$ The goal of preprocessing is to ensure that the coupling weights limits of the COBI hardware are respected. We focus on preprocessing for the COBI hardware with 45 physical spins, and 5-bit coupling weights in the range $[-14,+14]$^13^. Preprocessing techniques such as scaling, rounding, and truncation have been applied in previous work^11^. *Scaling* involves multiplying all couplings by a constant factor; *rounding* maps them to the nearest integers;and *truncation* clamps the couplings at the extreme values ($\pm 14$) allowed by the hardware. However, the direct use of these methods is not possible as the coupling values ($J_{ij}$ and $h_i$) for the IM Ising formulations span a wider dynamic range than 3SAT, as detailed in the Supplementary Materials. Using scaling to preserve large coupling coefficients would round the smaller values to zero, eliminating their influence. Using truncation would retain smaller values, but it clamps large ones to the hardware limits, which could change the optimization landscape. Both cases lead to a loss of precision that can degrade solver performance.

We first apply *scaling* to convert the fractional values introduced by the positional constant $c_i = 2^{-i}$ in (8) into integers. According to the distribution of coupling weights presented Fig. S3 in the Supplementary Materials, the coupling values for 10-node subgraphs, after applying relaxed penalty coefficients and scaling to integers, typically fall within the range $[-32, 20]$. We introduce the *mapping* step, where Ising spins with high coupling weights are mapped to one or more physical spins. The summation of all couplings between the mapped physical spins is used to represent the coupling of the original Ising spin variable. If Ising spin *i* were mapped to $m_i$ physical spins and Ising spin *j* to $m_j$ physical spins, there would be $m_i \times m_j$ physical couplings in total between the two groups, each of which may have a value of up to $\pm 14$. As a result, the effective coupling range between Ising spins *i* and *j* expands to $[-14m_im_j,14m_im_j]$, at the cost of using $m_i+m_j$ physical spins. In Fig. 3b, the coupling weights after scaling and rounding to integers are $J_{12}=-31$, $J_{23}=17$, which exceeds the hardware limit of $[-14, +14]$. By mapping the each spin variable in the formulation to two physical spins, the first to $(s_1,s_2)$ and second to $(s_3,s_4)$, i.e., $m_1=m_2=2$, as shown in the figure, the range of allowable coupling weights is extended to $[-56,56]$, supporting $J_{12} = -31$ and $J_{23}=17$. Thus, *scaling* and *mapping* are sufficient for our problem, and truncation is unnecessary.

Specifically, we apply graph decomposition to large graphs prior to preprocessing, producing subgraphs with configurations similar to the 10-node graphs. Since these 10-node benchmarks are known to be mappable to the COBI hardware, it is reasonable to assume that the resulting subgraphs after decomposition can also be successfully mapped and solved on COBI.

$\underline{\textit{Ising solution and solution interpretation}}$ The problem is then loaded to the COBI hardware, which returns the Ising solution and the corresponding optimal spins, $s_i, 1 \le i \le n$. In the solution interpretation step, the Ising solution is translated into an IM subgraph solution in the format of the seed set. Recall that the seed set corresponds to assignments of $s_v = 1$ in the QUBO formulation (8).

At the end of the Ising iteration, if the solution satisfies all constraints in Table 1, it is considered valid, and we run propagation (as described for the greedy search step above) from the seed set on the global graph. If the influence of this seed set is greater than the current IM solution, the IM solution is updated. If the solution does not satisfy all constraints, it is invalid, and the iteration ends without propagation, and the IM solution is not updated.

## Experiments and results

In this section, we will describe the results of our experiments on solving the LDAG problem for IM on COBI^13^, an Ising solver chip. We first overview our experimental setup, including LDAG IM benchmark generation, the baseline state-of-the-art IM solvers, and the metrics used to evaluate the solutions. Next, we evaluate various penalty coefficients to explore the most efficient formulation. Finally, we use the COBI Ising hardware solver to solve the benchmarks based on this efficient formulation and compare the results to other baseline solvers.

### Testcases, baselines, and metrics

We apply our techniques to six batches of LDAG IM testcases, corresponding to graphs with up to 200 nodes. The specifics of benchmarks in each batch, including the number of nodes, edges in the graph, their depth, and the optimal influence, are summarized in Table 5. All problems have 2-bit precision for the edge weights and node thresholds. Batches 0–4 consist of randomly generated graphs. Batches 2 and 4 are hard instances due to their higher depth compared to batches 1 and 3. A real-world social network, the Karate Club graph^24^, is used to build batch 5, with randomly assigned directions and weights to its edges. All these benchmarks and the source code to generate LDAG IM graphs are available in a public-domain repository^25^. We assume a seed set size, *k*, of two nodes for all IM benchmarks. The ground truth optimal solutions are determined by the Gurobi optimizer^26^. In the following sections, these benchmarks serve as the basis for evaluating the proposed formulation and the performance of the Ising solver. Specifically, batch 0, containing small benchmarks with 10 nodes, is used to explore Ising formulations. Batches 1–5 are used to evaluate the performance of the COBI solver against the following baseline IM solvers:

- **Simpath**^27^ is a path-based IM solver that estimates influence spread by enumerating all simple paths originating from candidate seed nodes. The algorithm begins with a vertex cover optimization, which identifies a small set of influential nodes using a heuristic to reduce the search space. It then applies a look-ahead optimization, iteratively assessing the influence spread from nodes within the vertex cover and those outside it separately.
- **IMM**^28^ is a near-linear time IM solver that uses martingales^29^ to handle dependencies during the sampling of reverse reachable (RR) sets, which represent the set of nodes that could influence a target node. It iteratively generates RR sets, then uses the standard greedy algorithm for maximum coverage with RR sets to optimize the influence.
- **qbsolv**^30^ is a software framework that employs the Tabu search algorithm^31^ to solve QUBO problems with floating-point precision. It decomposes the original QUBO into a set of smaller subQUBOs, each of which is iteratively solved using Tabu search until a global solution converges. The qbsolv method also requires decomposition, similar to COBI, and uses a workflow similar to Fig. 3.

To evaluate the performance of the IM solvers, we define the following metrics:

- **Number of iterations**: For the loops in Fig. 3a,b, we report the number of times the loop is executed. This metric is only used for qbsolv and COBI, which employ this workflow.
- **Influence ratio**: We define this as the ratio of $\sigma _{G,M}(S)$, the influence of the solution to (1), to $\sigma _{G,M}(S^*)$, the ground truth optimal influence. This metric reflects the quality of the solution obtained by each method.
- **Time-to-solution (TTS) and Energy-to-solution (ETS)**: The overall execution time and energy, respectively, required by an IM solver to solve a benchmark.

The Simpath solver is deterministic and therefore has deterministic performance metrics, and its TTS and ETS are the solver runtime and energy, respectively. In contrast, IMM, qbsolv, and COBI are stochastic solvers whose solution and performance vary from run to run. To ensure a fair comparison, these stochastic solvers are executed multiple times, and we apply maximum likelihood estimation (MLE)^32,33^ to have a stable estimation of their runtime to compute TTS based on observed outcomes. Specifically, for qbsolv and COBI, the runtime for the $i^{th}$ stochastic run is defined as $\hbox {Runtime}_{\text {per-run}} =$ $N_{iter,i} \times t_{iter,i}$, where $N_{iter,i}$ and $t_{iter,i}$ are, respectively, the number of iterations and the solver runtime per iteration. As for MLE, we define success as obtaining *optimal influence probability*, $p_{\text {OI}} = p(\sigma _{G,M}(S)=\sigma _{G,M}(S^*))$, which denotes the probability that a solution achieves the optimal influence in a single run. Then, elementary probability theory states that the probability of achieving an optimal solution after $\nu$ independent runs is $(1 - (1 - p_{\text {OI}})^{\nu })$. Thus, for the stochastic solvers, the runtime corresponds to the cumulative cost of these repeated runs, which is a random quantity. However, we can compute the expected time required to achieve the solution with a specified probability, $p_{\text {target}}$, after repeated runs. To evaluate this metric, we employ a “time-to-solution” (TTS) metric based on an established technique from the literature^34^:

11 $$\begin{aligned} \text {TTS} =\frac{\ln (1-p_{\text {target}})}{\ln (1-p_{\text {OI}})} \times \frac{1}{\nu }\sum _{i=1}^{\nu } \text {Runtime}_i \end{aligned}$$

where $\hbox {Runtime}_i$ is the runtime of the $i^{\textrm{th}}$ independent run.

The ETS for a stochastic or deterministic solver is defined as the total energy consumed to achieve a probability of $p_{\text {target}}$, reflecting the energy efficiency of the solver. Based on the above definitions of the TTS, the ETS is given by $\text {TTS} \times P_{solver}$, where $P_{solver}$ represents the power dissipation of the hardware platform (Ising solver or CPU) on which the solver is run.

### Formulation exploration

COBI and qbsolv are Ising-based IM solvers that require a QUBO/Ising formulation in their workflow. As discussed in “Mapping the LDAG influence model to a hardware Ising solver”, the penalty coefficients in formulation (8) must be tuned to use the physical spins efficiently on the Ising solver. Therefore, we evaluate how different values of penalty coefficients impact the overall performance of the LDAG IM formulation and assess solution quality under various penalty settings. We use the qbsolv framework to determine these settings.

**Table 4:** Simulation results for various values of the penalty coefficients on all benchmarks in batch 0.

$\lambda _1=\lambda _2$	$\lambda _3$	Number of physical spins (mean)	$p_{C}$	Influence ratio	$p_{OI}$	Influence ratio per physical spin	$\lambda _1=\lambda _2$	$\lambda _3$	Number of physical spins (mean)	$p_{C}$	Influence ratio	$p_{OI}$	Influence ratio per physical spin
2	8	41.4	0.15	0.15	0.15	0.0036	8	8	52.0	0.14	0.14	0.13	0.0027
2	16	41.7	0.35	0.35	0.34	0.0084	8	16	52.0	0.41	0.41	0.39	0.0078
2	32	46.8	0.63	0.63	0.59	0.0134	8	32	53.0	0.91	0.90	0.87	0.0171
2	64	62.1	0.57	0.55	0.48	0.0089	8	64	63.9	1	0.98	0.88	0.0153
2	128	79.2	0.55	0.48	0.28	0.0061	8	128	77.9	1	0.97	0.84	0.0125
4	8	51.0	0.17	0.17	0.17	0.0033	16	8	63.0	0.14	0.14	0.13	0.0022
4	16	50.7	0.43	0.43	0.43	0.0085	16	16	63.0	0.42	0.40	0.33	0.0064
4	32	49.7	0.87	0.86	0.83	0.0174	16	32	63.6	0.87	0.86	0.79	0.0135
4	64	62.4	0.99	0.96	0.85	0.0155	16	64	74.3	1	1.00	0.98	0.0134
4	128	77.6	0.98	0.92	0.71	0.0118	16	128	84.4	1	0.98	0.89	0.0116

Table 4 presents the results obtained without decomposition and using various penalty coefficients $\lambda _i$ chosen as powers of 2 to minimize errors under fixed-point precision. To focus on the penalty function formulation, our testcases correspond to the 100 benchmarks in batch 0, which are small enough that they do not require decomposition on the stochastic solvers. The reults for 50-node benchmarks in batch 1 are introduced in Table S1 in the Supplementary Materials. As in (7), we assume $\lambda _1=\lambda _2$. The mean number of physical spins indicates the number of spins required to implement the Ising formulation in (8) after preprocessing: notice that this varies because, depending on the coupling weights, a variable may be mapped to more than one physical spin. The constraint satisfaction probability, $p_C$, indicates the fraction of solutions in which the solution satisfies all constraints in (1). Any invalid solution that does not satisfy all constraints is assumed to have an influence ratio of zero. Then we evaluate the influence ratio, and the optimal influence probability, $p_{\text {OI}}$, reporting their means over $\nu =100$ repeated solutions for each benchmark.

The results show that as expected, the constraint satisfaction probability $p_C$ increases with higher values of $\lambda _i$, because these higher values place a larger focus on the constraints. However, both the influence ratio and $p_{\text {OI}}$ initially increase and then decrease as the $\lambda _i$s increase. The latter trend occurs because, as larger penalty coefficients increasingly dominate the objective function, the relative contribution of the original objective term of influence maximization is diminished. The table also shows the influence ratio per physical spin, defined as the influence ratio divided by the number of physical spins. Since the number of physical spins represents the hardware resource, a higher influence ratio per physical spin indicates either greater performance under the same hardware constraints or equivalent performance using fewer hardware resources. Therefore, we use it to quantify the efficiency of the formulation. We find that among the tested penalty coefficients, setting $\lambda _{1} = \lambda _{2} = 4$, and $\lambda _{3} = 32$ yields the highest efficiency. We use this assignment in the formulation in all subsequent experiments.

### Results on IM testcases

The optimization formulation, with the empirically chosen values of the penalty coefficients, is applied to our workflow in Fig. 3a to solve the LDAG IM problem using the COBI Ising hardware solver^13^. Our testcases consist of all benchmarks in batches 1–5 in Table 5, with $\nu = 100$ independent runs per benchmark used to obtain the influence ratio and $p_{\text {OI}}$.

**Table 5:** A description of the set of LDAG benchmarks used in this work.

Batch	# graphs	# nodes per graph	#edges per graph	Mean depth ofgraphs	Optimal influence given a seedset of 2			
					Max	Min	Mean	Median
0	100	10	5–17	2.64	10	3	5.6	5
1	100	50	157–178	7.22	50	7	35.25	38.5
2	100	50	142–168	11.6	50	5	30.43	32
3	100	200	764–780	6.60	200	26	148.56	158.5
4	100	200	665–696	20.5	200	6	109.52	108
5 (Karate Club)	10	34	78	5.8	27	8	15.8	13.5

**Fig. 4:**
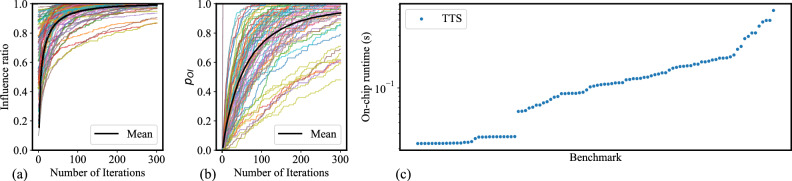
IM results with COBI on batch 1 (**a**) Influence ratio, (**b**) Optimal influence probability, $p_{\text {OI}}$, and (c) TTS.

Fig. 4a,b illustrate, across all benchmarks in batch 1, the improvement in the influence ratio and $p_{\text {OI}}$, respectively, as the 100 independent runs proceed. Each curve in the figure represents one benchmark, except for the black curve, which represents the mean value. The influence ratio is 0.94 over 100 iterations, and 92% testcases can achieve $p_{\text {OI}} \ge 0.7$ within 300 iterations. Based on the measured runtime of COBI, we compute the TTS for each benchmark in batch 1, using MLE to determine the optimal influence probability, and setting $P_{\text {target}} = 0.95$. The sorted TTS data is shown in Fig. 4c, where each point on the scatter plot represents the TTS for an IM benchmark, corresponding to a curve in Fig. 4a,b. The TTS values range from 0.02s to 0.78s. Some benchmarks on the left of the sorted TTS plot exhibit very low TTS because their initial greedy solutions (where each of the influenced nodes is activated by a single seed) already achieve the optimal influence, and the stopping criterion is met at an early stage with a smaller number of iterations. In contrast, benchmarks with higher TTS are harder problems that require more iterations to solve, and include nodes that are activated due to propagated influence from all seeds.

**Fig. 5:**
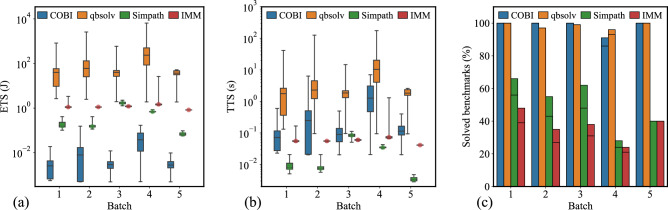
(**a**) Batch-wise ETS distribution, (**b**) Batch-wise TTS distribution, (**c**) Batch-wise percentage of solved benchmarks.

Similarly, we generate the runtime for COBI and three baseline IM solvers, qbsolv, Simpath, and IMM, on batch 1 through batch 5 in Table 5. After collecting the results, we categorize the benchmarks in each batch into the solved and the unsolved testcases. For Simpath, it is based on whether it can provide a solution with optimal influence. For stochastic solvers (COBI, qbsolv and IMM), a benchmark is classified as solved if the stochastic solver can provide a solution with optimal influence in at least one of the runs, then $p>0$ in (11). Otherwise, it is categorized as unsolved. Among the solvers, COBI and qbsolv, both stochastic methods, are found to solve more benchmarks across all batches, while the two classical IM solvers (Simpath and IMM) exhibit a higher percentage of unsolved benchmarks. IMM has the highest percentage of unsolved benchmarks. The instances in batches 2 and 4 are more challenging and have more unsolved benchmarks.

**Table 6:** A comparison of COBI vs. software solvers for IM, showing the percentage of solved benchmarks, and statistics of the TTS and ETS over all benchmarks in batch 1 through batch 5.

Batch	Solver	Solved benchmarks (%)	TTS (s)			ETS (J)		
			Mean	Median	S.D.	Mean	Median	S.D.
1	COBI	100	0.1388	0.1061	0.1425	0.0033	0.0025	0.0034
	qbsolv	100	4.1335	2.0025	8.1218	82.670	40.051	162.44
	Simpath	56	0.0097	0.0078	0.0039	0.1948	0.1556	0.0789
	IMM	39	0.0579	0.0556	0.0179	1.1579	1.1115	0.3586
2	COBI	100	0.6718	0.3253	1.1560	0.0161	0.0078	0.0277
	qbsolv	97	8.4033	3.0049	20.237	168.07	60.097	404.73
	Simpath	43	0.0087	0.0074	0.0035	0.1740	0.1479	0.0699
	IMM	27	0.0553	0.0559	0.0014	1.1068	1.1178	0.0288
3	COBI	100	0.1241	0.1197	0.0774	0.0030	0.0029	0.0019
	qbsolv	99	2.7808	1.9963	4.3706	55.615	39.925	87.414
	Simpath	48	0.0845	0.0839	0.0100	1.6898	1.6786	0.1991
	IMM	31	0.0596	0.0599	0.0025	1.1925	1.1981	0.0498
4	COBI	86	2.0865	1.5578	2.0284	0.0501	0.0374	0.0487
	qbsolv	93	31.646	11.805	58.021	632.91	236.10	1160.4
	Simpath	24	0.0346	0.0343	0.0022	0.6927	0.6870	0.0446
	IMM	21	0.1311	0.0734	0.2683	2.6211	1.4677	5.3656
5	COBI	100	0.1371	0.1148	0.1102	0.0033	0.0028	0.0026
	qbsolv	100	1.7165	1.8288	0.8178	34.330	36.577	16.357
	Simpath	40	0.0035	0.0033	0.0008	0.0710	0.0666	0.0170
	IMM	40	0.0411	0.0412	0.0005	0.8225	0.8236	0.0109

Next, we compute the TTS and ETS for the solved benchmarks in each batch on COBI, summarizing their mean, median, and standard deviation in Table 6. As shown in Fig. 5a, COBI’s ETS is significantly lower than that of all other solvers across all benchmark batches. This advantage stems primarily from the COBI chip’s much lower typical power consumption (24 mW) compared to a CPU (20 W). For Simpath and IMM in Fig. 5a,b, certain boxplot distributions appear very narrow on the log-scale plots; to enhance visibility, we overlay a circle marker at the median values. Specifically, COBI achieves energy consumption reductions of 3–4 orders of magnitude relative to qbsolv, and 1–2 orders of magnitude compared to Simpath and IMM. These results demonstrate a substantial improvement in energy efficiency enabled by the COBI-based influence maximization solver. Importantly, these TTS and ETS trends remain consistent across both the synthetic benchmark batches and the real-world Karate Club dataset.

Additionally, the reported TTS and ETS metrics account only for the runtime and energy of the COBI hardware in iterations. The digital overhead, including greedy search, decomposition, and preprocessing, is treated separately:

- **Greedy search:** The runtime of the greedy search is included in the TTS. It is executed only once with linear complexity in node count, yielding a constant overhead independent of the number of iterations.
- **Decomposition:** The decomposition time is minimal (typically $<100\,\mu \text {s}$) and negligible relative to the Ising solver’s runtime. It is essentially an early-terminated BFS traversal with linear complexity in the subproblem size, and can be further accelerated via ASIC or FPGA implementations.
- **Preprocessing:** Currently implemented in software, preprocessing involves iterating over all coupling weights and can be slow. In future work, we plan to implement it on hardware (e.g., ASIC or FPGA) to work in a pipelined fashion with COBI, effectively eliminating its contribution to TTS.

Typical runtime and energy measurements for both software and hardware preprocessing are provided in the Table S2 in the Supplementary Materials. On a log-scale ETS plot, the additional energy from hardware-based preprocessing remains within the same order of magnitude as COBI.

The TTS distributions are plotted in Fig. 5b, where the whiskers represent the maximum and minimum values, and the boxes indicate the first and third quartiles. COBI can achieve one order of magnitude lower TTS compared to qbsolv. Although both solvers use the Ising formulation to address the IM problem, the significant improvement comes from COBI’s speed advantage. By leveraging the physical dynamics of oscillators, COBI outperforms CPU-based mathematical optimization heuristics, as discussed in “Hardware Ising solver”. On the surface, COBI does not appear to exhibit superior TTS compared to Simpath and IMM. However, since TTS is calculated only on the solved benchmarks, it is important to note that Simpath and IMM have a significantly higher proportion of unsolved benchmarks, as shown in the third column of Table 6. Therefore, the overall TTS performance for COBI is stronger than Simpath or IMM, as it successfully solves many more benchmarks.

**Fig. 6:**
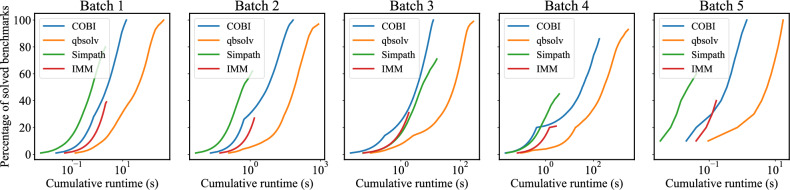
Cumulative runtime based on TTS for all benchmarks in batch 1 through batch 5.

To validate the robustness of our conclusions, we reassess the solvability analysis under a relaxed criterion: instead of considering a benchmark as solved when it achieves an influence ratio of 1, i.e., the optimal influence, we now consider labeling it as solved if it is within 10% of the optfigsimal solution, i.e., its influence ratio is $\ge 0.9$. Fig. 5c presents the percentage of solved benchmarks in each batch under this relaxed criterion. For clarity, each bar includes a marker that shows the proportion of benchmarks that achieved an influence ratio equal to 1. A fully solid bar with no marker indicates that an influence ratio of 1 is achieved (and therefore, the solutions automatically satisfy the relaxed criterion of $\ge 0.9$ too). From the plot, it is evident that while Simpath and IMM exhibit slight improvements in solvability for batches 1–4 under the relaxed threshold, their overall performance continues to remain substantially lower than that of COBI and qbsolv, as reflected by the shorter green and red bars compared to blue and orange. Therefore, the conclusion that COBI outperforms classical IM solvers in terms of solvability remains valid even if the optimality criterion is relaxed. The corresponding TTS and ETS plots under the relaxed criterion are provided in Table S3 and Fig. S4 in the Supplementary Materials.

It is important to note that the lower solvability of Simpath and IMM in our experiments does not imply that these solvers are ineffective. IM is an NP-hard problem; heuristic-based solvers Simpath and IMM are designed to handle large-scale graphs on conventional CPUs efficiently. To achieve polynomial or near-linear runtimes, these solvers trade off some degree of solution accuracy and performance^27,28^. Nonetheless, their runtime advantages and scalability on CPUs make them valuable for many practical large-scale IM applications.

To better illustrate both solvability and TTS across benchmarks, we sort the TTS values in ascending order for each batch. Assuming that the benchmarks are solved sequentially in this order, we compute the cumulative runtime for each batch and plot it against the percentage of solved benchmarks, as shown in Fig. 6. For benchmarks with no more than 50 nodes (batches 1, 2, and 5), COBI is not the fastest on the easier benchmarks, but can solve harder benchmarks, as reflected by the highest value in its curve. For the larger 200-node benchmarks in batches 3 and 4, Simpath and COBI perform similarly on easier problems, and COBI successfully solves more hard benchmarks than Simpath and IMM.

We report the runtime and energy consumption of Gurobi^26^ when solving the benchmark batches on a CPU, as shown in Table 7. As a commercial solver, Gurobi is highly optimized for CPU performance and leverages advanced heuristics and historical solution databases to speed up computation. Consequently, the reported runtime may not accurately represent the time needed to solve an IM benchmark from scratch, making direct comparisons with other IM solvers potentially unfair, particularly with those implemented in Python. These results are intended as a general reference for evaluating solver efficiency. Despite this, our Ising-based IM solver demonstrates significantly lower energy consumption, as indicated by the energy values in Table 7 and ETS in Table 6.

**Table 7:** Runtime for Gurobi on batches 1–5.

Batch		1	2	3	4	5
Runtime (s)	Mean	0.033	0.061	0.109	0.348	0.040
	Median	0.025	0.038	0.074	0.274	0.019
Energy (J)	Mean	0.66	1.22	2.18	6.96	0.80
	Median	0.50	0.76	1.48	5.48	0.38

## Conclusion

This work presents a workflow for solving the linear threshold model of IM on directed acyclic graphs using a CMOS Ising solver, providing a practical example and insights for tackling constrained optimization problems on Ising hardware. We derive an Ising formulation of IM through integer linear programming, employ decomposition to efficiently handle larger graphs, and utilize a CMOS solver, COBI^13^, to solve the Ising formulations. The workflow incorporates formulation, decomposition, and preprocessing steps to effectively map the Ising formulation onto the COBI hardware. Performance comparisons among COBI, qbsolv, and two classical IM solvers, Simpath and IMM, highlight that the COBI-based approach has a higher ability to solve IM on randomly generated benchmarks with 50 and 200 nodes and the real-world benchmark, the Karate Club graph. In addition, the COBI-based approach demonstrates 2–3 orders of magnitude lower energy consumption than qbsolv, which is based on a software Tabu sampler; 1–2 orders of magnitude lower energy than other classical IM solvers Simpath and IMM. The results demonstrate the potential of Ising solvers for energy-efficient IM applications.

## Supplementary Information

Supplementary Information.

## Data Availability

The datasets used and/or analyzed during the current study are available from the corresponding author on reasonable request.
