# Supplementary material for: Influence maximization under the linear threshold model on a CMOS Ising solver
Source: Sci Rep. 2025 Dec 7;15:43604. doi: 10.1038/s41598-025-27169-5 (PMC12698711; doi:10.1038/s41598-025-27169-5)
Supplement: Supplementary file 1 — Supplementary Information. [file 41598_2025_27169_MOESM1_ESM.pdf]

Supplementary Materials: Influence Maximization under the Linear Threshold Model on a CMOS Ising Solver

We provide extended data to support and expand on the main text of the manuscript. Sec. S1 analyzes the performance of BFS and improved BFS decomposers. Sec. S2 presents the distribution of coupling weights used in preprocessing, supporting the choice of scaling and mapping discussed in Sec. 4. Sec. S3 then shows the Ising formulation for 50-node benchmarks, motivating the need for graph decomposition. Sec. S4 provides typical runtime and power for CPU, ASIC, and FPGA. Sec. S5 provides detailed TTS and ETS results under our relaxed success criterion, complementing the optimal influence ratio definition in Sec. 5.

S1 Decomposer exploration in graph decomposition

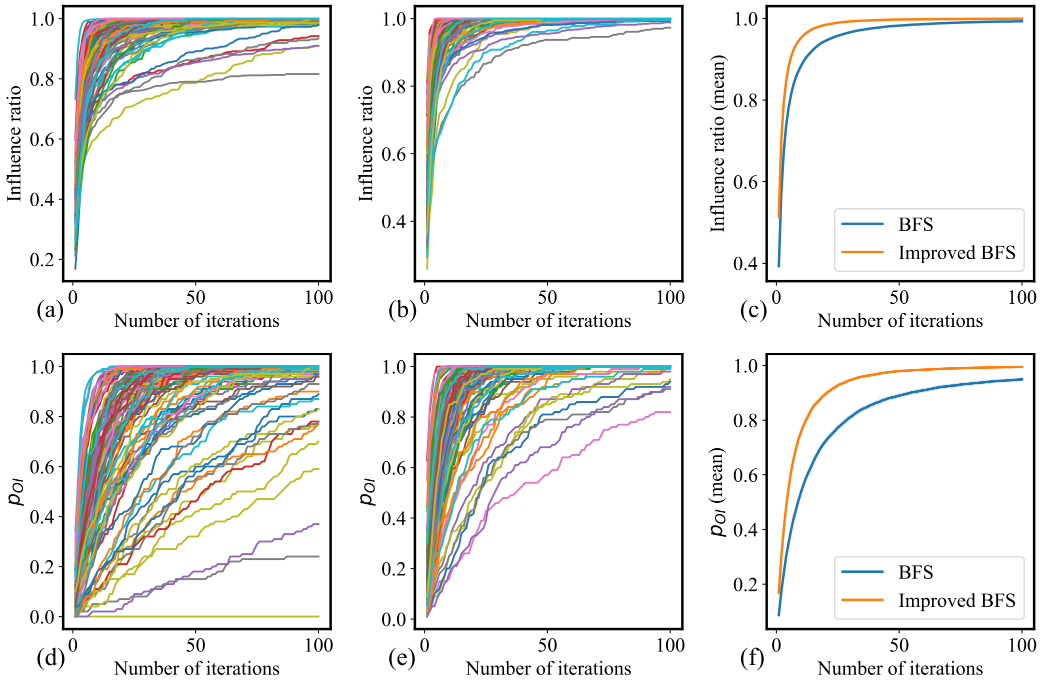

Figure S1. Influence ratio with (a) BFS decomposer, (b) Improved BFS decomposer; (c) Mean influence ratio. Optimal influence probability (p_{OI}) with (d) BFS decomposer, (e) Improved BFS decomposer; (f) Mean optimal influence probability.

In Sec. 4, we introduced the BFS decomposer and the improved BFS decomposer. Figure S1 displays various metrics, previously defined in Sec. 5.1, for these two decomposers across 100 benchmarks in batch 1 in Table 3. In Figure S1(a) and (c), each curve represents the influence ratio for the BFS and improved BFS decomposer,

respectively, as a function of the number of iterations for a particular graph. Figure S1(d) and (e) shows a similar visualization for the two decomposers, where each curve represents the optimal influence probability, p_{OI}. In some cases, the BFS decomposer achieves a high influence ratio but fails to reach a high probability of matching the optimal influence. This is because in cases where some nodes have a high degree, the BFS decomposer may generate subgraphs with a shallow depth. The mean curve for the influence ratio and the optimal influence probability for both decomposers, across all benchmarks, is shown in Figures S1(c) and (f), respectively. From these results, it is seen that overall, the improved BFS decomposer shows better performance in both the influence ratio and the optimal influence probability.

Having confirmed the superiority of the improved BFS decomposer, we further investigate the impact of the tunable parameter γ. As described in Sec. 4, unlike the BFS decomposer, which adds all unvisited neighbors of a node to the queue, the improved BFS decomposer randomly selects at most γN_{sub} neighbors, where $0 < \gamma \leq 1$. To assess the effect of this parameter, we vary γ and evaluate the resulting performance on COBI hardware. Figure S2 presents the influence ratio and optimal influence probability for different values of γ. Among the tested settings, $\gamma = 0.3$ yields the best performance. Therefore, all graph decompositions in Sec. 5.3 are performed using the improved BFS decomposer with $\gamma = 0.3$.

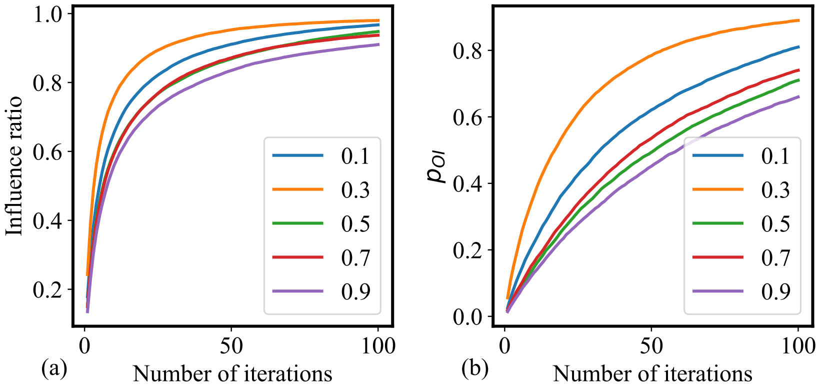

Figure S2. Performance of different γ on COBI, (a) Influence ratio, (b) Optimal influence probability p_{OI}.

S2 Distribution of coupling weights in preprocessing

As discussed in the workflow in Sec. 4, each iteration begins with generating an Ising formulation after graph decomposition, followed by preprocessing to produce a hardware-compatible Ising formulation for COBI. The Ising formulation contains floating-point coupling weights (h_i and J_{ij}), but COBI[1] supports only integer weights within $[-14, +14]$. To explore the preprocessing, we use the most efficient IM problem formulation with penalty coefficients $\lambda_1 = \lambda_2 = 4$, $\lambda_3 = 32$ based on Table 3. We scale the floating-point coupling weights in the Ising formulation to integers, for all benchmarks in batch 0, we plot their distribution in Figure S3. The x-axis represents coupling weights after scaling, while the y-axis indicates their frequency of occurrence as a percentage. The maximum positive coupling weight is 20, and the minimum is -32.

Given this distribution, if we normalize the coupling weights by scaling the maximum absolute value ($|-32|$) to fit within COBI's limits, approximately 5.4% of the weights would require rounding, leading to precision loss. Alternatively, truncating values outside the $[-14, +14]$ range would affect 7.2% of the weights, which is also undesirable. To address this, we introduce mapping to preprocessing to preserve precision. By mapping one Ising spin to two physical spins on COBI, the supported range of coupling weights would be $[-56, 56]$ $(2 \times 2 \times 14 = 56)$, allowing all weights to be represented without truncation. Therefore, combining scaling with mapping enables accurate and hardware-compatible Ising formulations for COBI.

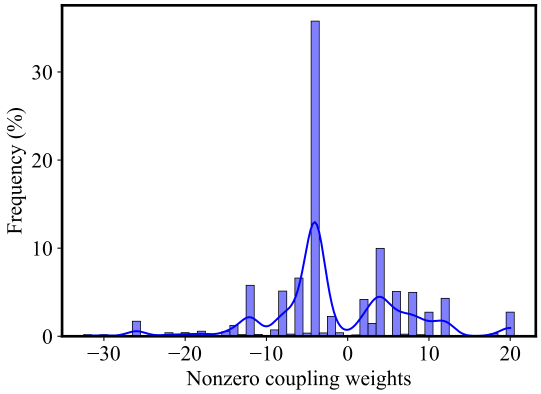

Figure S3. Distribution of coupling weights of Ising formulation over 100 benchmarks in batch 1.

S3 Ising Formulation of 50-node graph

In Sec. 5, we present formulation exploration on 10-node benchmarks from batch 0. Following the same methodology, we evaluate the formulation on 50-node benchmarks from batch 1 using qbsolv[2] as the Ising solver and report the mean evaluation metrics in Table S1. Compared to the 10-node results in Table 3, all evaluation metrics, including the number of physical spins, the constraint satisfaction probability p_C, influence ratio, and the optimal influence probability p_{OI}, consistently degrade across penalty coefficient settings. These results suggest that the accuracy of qbsolv is insufficient to ensure solution quality for larger Ising formulations and that problem decomposition is essential for qbsolv.

λ_1	λ_2	λ_3	Number of physical spins	p_C	Influence ratio	p_{OI}
16	8	64	608.7	0.173	0.163	0.141
16	8	128	664.3	0.246	0.197	0.159
16	8	256	787.7	0.266	0.142	0.099
16	8	512	986.9	0.327	0.119	0.074
16	16	64	608.6	0.185	0.163	0.141
16	16	128	664.1	0.283	0.204	0.159
16	16	256	787.7	0.314	0.153	0.101
16	16	512	986.6	0.456	0.137	0.080
16	32	64	608.5	0.170	0.163	0.141
16	32	128	663.3	0.204	0.192	0.162
16	32	256	788.0	0.402	0.162	0.100
16	32	512	977.1	0.660	0.146	0.073

Table S1. Simulation results for relaxed penalty coefficient on 50-node benchmarks in batch 1.

Furthermore, given that COBI only supports integer coupling weights (unlike qbsolv, which can use floating-point weights) and solves the same underlying problem, it is reasonable to expect that the quality of a COBI solution would also degrade without partitioning. This observation further motivates the integration of graph decomposition in our COBI-based IM workflow, enabling the solver to handle large graphs by partitioning them into smaller, tractable subgraphs. However, given the hardware limitations of COBI, it is not possible to perform direct experiments to directly validate this hypothesis on larger partition sizes.

S4 Typical runtime and power for preprocessing

In Sec. 4, we introduce preprocessing as an essential component of our workflow to ensure high performance. To assess the runtime and energy efficiency of the complete system, we analyze the preprocessing runtime of the Ising formulation on COBI using 10-node problems in batch 0, as reported in Table 5, which corresponds to the COBI configuration after decomposition.

Currently, preprocessing is implemented in software on a CPU (Intel Xeon Gold 6240R, 2.40 GHz). Because each coupling weight is processed sequentially on a single core, this approach is slow due to the iterative looping over all coupling weights. To overcome this limitation, we plan to migrate preprocessing to hardware, either by deploying it on the Xilinx Artix-7 FPGA already integrated on the COBI board or by embedding it directly into the ASIC (28 nm technology). This hardware integration enables preprocessing to operate in parallel with the COBI core. Under this assumption, we estimate the runtime and power for both FPGA- and ASIC-based implementations, as summarized in Table S2.

Task	Implementation	Runtime (ms)	Power (mW)	Energy(mJ)
Preprocessing	Software (Python on CPU)	2.0	20,000	400
	FPGA (projected)	0.2	50	0.01
	ASIC (projected)	0.1	10	0.001
Ising solving	COBI hardware	0.4	24	0.0096

Table S2. Typical runtime and power for preprocessing under software and (projected) hardware implementations.

For **TTS** computation, hardware-based preprocessing can be fully pipelined with the Ising solver, making its contribution negligible. In contrast, software preprocessing would dominate TTS due to its significantly longer runtime.

For **ETS** computation, the difference between software and hardware implementations spans several orders of magnitude. In a software setup, ETS is dominated by preprocessing energy (runtime $\times$ CPU power). In a hardware setup, preprocessing energy is comparable to that of the Ising solver itself. Since ETS is plotted on a logarithmic scale, adding the constant hardware preprocessing energy does not affect the overall trends or conclusions.

S5 TTS and ETS under a relaxed criterion

In Sec. 5, we evaluate solvability under two success criteria: the optimality criterion, i.e., influence ratio = 1, and a relaxed criterion, i.e., influence ratio ≥ 0.9. In Sec. 5, it is demonstrated in Figure 4(c) that the relaxed criterion does not significantly improve the percentage of benchmarks solved by SIMPATH and IMM. However, due to space constraints, only the TTS and ETS results under the optimality criterion are reported in Sec. 5. In this section, we expand the analysis to include complete results under the relaxed criterion. Table S3 summarizes the percentage of unsolved benchmarks, along with TTS and ETS metrics for batches 1–5. The corresponding distributions are visualized in Figure S4.

Comparing these results with those under the optimality criterion (Table 5 and Figure 3(a), (b)), all solvers show modest improvements in both TTS and ETS when the relaxed criterion is applied. However, the key conclusions from the optimality criterion remain consistent under the relaxed criterion. Specifically, COBI continues to demonstrate 3–4 orders of magnitude lower energy consumption than qbsolv, and 1–2 orders of magnitude lower than SIMPATH and IMM. Moreover, COBI achieves approximately one order of magnitude lower TTS compared to qbsolv, while solving a similar number of benchmarks. Since TTS is calculated only over solved benchmarks, the percentage of solved benchmarks is crucial. As shown in the third column of Table S3, SIMPATH and IMM show slight gains in solvability under the relaxed criterion, but still exhibit a significant gap compared to COBI and qbsolv. These observations reinforce our conclusion from Sec. 5: COBI is the most energy-efficient solver across a broad set of benchmarks, driven by higher solvability and lower runtime.

Batch	Solver	Solved Benchmarks (%)	TTS (s)			ETS (J)		
			Mean	Median	S.D.	Mean	Median	S.D.
1	COBI	100	0.1033	0.0718	0.1110	0.0025	0.0017	0.0027
	qbsolv	100	3.2320	1.7636	7.2345	64.640	35.273	144.69
	SIMPATH	66	0.0223	0.0139	0.0214	0.4453	0.2772	0.4274
	IMM	48	0.0575	0.0557	0.0161	1.1494	1.1136	0.3230
2	COBI	100	0.5143	0.2470	0.9706	0.0123	0.0059	0.0233
	qbsolv	97	6.3022	2.2892	17.393	126.04	45.783	347.86
	SIMPATH	55	0.0181	0.0076	0.0219	0.3628	0.1526	0.4381
	IMM	35	0.0552	0.0557	0.0014	1.1036	1.1140	0.0274
3	COBI	100	0.1054	0.0898	0.0772	0.0025	0.0022	0.0019
	qbsolv	99	2.1159	1.8531	2.1812	42.318	37.063	43.624
	SIMPATH	62	0.1827	0.0895	0.1988	3.6542	1.7901	3.9762
	IMM	38	0.0599	0.0604	0.0025	1.1986	1.2077	0.0491
4	COBI	91	1.7725	1.2670	1.8537	0.0425	0.0304	0.0445
	qbsolv	96	21.366	10.375	35.132	427.32	207.50	702.63
	SIMPATH	28	0.0783	0.0387	0.0850	1.5653	0.7743	1.7010
	IMM	24	0.1239	0.0734	0.2509	2.4773	1.4683	5.0186
5	COBI	100	0.1371	0.1148	0.1102	0.0033	0.0028	0.0026
	qbsolv	100	1.7165	1.8288	0.8178	34.330	36.576	16.357
	SIMPATH	40	0.0069	0.0036	0.0059	0.1375	0.0723	0.1177
	IMM	40	0.0411	0.0412	0.0005	0.8225	0.8236	0.0109

Table S3. A comparison of COBI vs. software solvers for IM, showing the percentage of solved benchmarks, and statistics of the TTS and ETS under the relaxed criterion (influence ratio ≥ 0.9) over all benchmarksin batch 1 through batch 5.

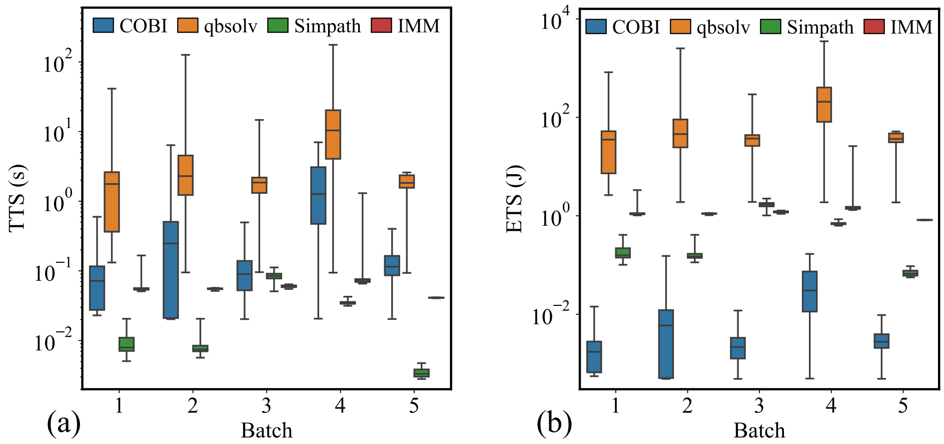

Figure S4. (a) Batch-wise TTS distribution, (b) Batch-wise ETS distribution, under the relaxed criterion (influence ratio$\geq$ 0.9).

References

1. Lo, H., Moy, W., Yu, H., Sapatnekar, S. & Kim, C. H. An Ising solver chip based on coupled ring oscillators with a 48-node all-to-all connected array architecture. *Nat. Electron.* **6**, 771–778 (2023).

2. D-Wave qbsolv (2018). https://github.com/dwavesystems/qbsolv.
